# Integrated metabolomic and transcriptomic analysis identifies enhanced phosphatidylcholine metabolism in placenta accreta spectrum

**DOI:** 10.1186/s12884-026-09552-w

**Published:** 2026-06-23

**Authors:** Zhenping Liu, Xijiang Liu, Wenxia Zhang, Chenggong Sun, Yuyan Ma, Hongyan Wang

**Affiliations:** 1 Department of Obstetrics and Gynecology, Qilu Hospital of Shandong University, Jinan, 250001 China; 2 Department of Anesthesiology, Shandong Provincial Hospital Affiliated to Shandong First Medical University, Jinan, 250021 China

**Keywords:** Placenta accreta spectrum (PAS), Metabolomics, Transcriptomics, Glycerophospholipid metabosim, Lipid metabolism

## Abstract

**Background:**

Placenta accreta spectrum (PAS) is a serious obstetric complication with limited understanding of its molecular basis. This study investigates metabolic and transcriptomic alterations in PAS placentas to uncover potential mechanisms and biomarkers.

**Methods:**

Placental tissues from PAS patients (*n* = 19) and controls (*n* = 10) underwent untargeted metabolomics via Liquid chromatography–tandem mass spectrometry (LC-MS/MS). Differential metabolites were analyzed for pathway enrichment. RNA sequencing was performed to identify differentially expressed genes (DEGs), followed by Kyoto Encyclopedia of Genes and Genomes (KEGG) and disease ontology enrichment. Integrated metabolomic-transcriptomic analysis was used to identify key metabolic pathways, and findings were validated in an expanded cohort (22 PAS cases, 50 controls). Receiver operating characteristic (ROC) curve analysis was performed to assess the diagnostic performance of Lecithin, phosphate cytidylyltransferase 1 beta (PCYT1B), and their combination for predicting PAS.

**Results:**

A total of 203 metabolites were differentially expressed in PAS placentas, with lipid and lipid-like molecules comprising the largest class. Several phosphatidylcholine (PC) species were significantly upregulated, indicating dysregulated glycerophospholipid metabolism. Transcriptome analysis identified 578 DEGs, including upregulation of PCYT1B and endothelial lipase (LIPG), both involved in lipid metabolism. Enrichment analysis revealed activation of glycerolipid and glycosphingolipid pathways and the potential involvement with metabolic disorders. Integrated analysis highlighted enhanced phosphatidylcholine biosynthesis, supported by elevated PCYT1B expression and increased total PC levels in PAS placentas. Lecithin showed higher sensitivity but lower specificity, while PCYT1B displayed intermediate performance. Their combination yielded the best discrimination with an area under the curve (AUC) of 0.791 (95% CI: 0.684–0.899).

**Conclusions:**

PAS is characterized by significant alterations in lipid metabolism, particularly increased phosphatidylcholine biosynthesis via the Kennedy pathway. These findings provide new insights into the molecular pathogenesis of PAS and suggest potential metabolic targets for early diagnosis or intervention.

**Supplementary Information:**

The online version contains supplementary material available at https://doi.org/10.1186/s12884-026-09552-w.

## Introduction

Placenta implantation, the process by which the developing embryo attaches to and embeds itself into the lining of the uterus (endometrium) during early pregnancy, is a critical physiological process establishing a connection between the developing embryo and the maternal uterine wall [1]. During implantation, trophoblasts burrow into the maternal blood vessels establishing a connection between the fetal and maternal circulatory systems allowing for the efficient transfer of nutrients and oxygen to support fetal growth, the removal of waste products, and the production of hormones essential for pregnancy maintenance [1].

When normal placental implantation occurs, trophoblasts invade the endometrium to an appropriate depth and in the proper location, the upper portion of the uterine cavity. In contrast, abnormalities in implantation may lead to serious obstetric complications, such as placenta previa and placenta accreta spectrum (PAS) disorders [2]. Placenta previa develops when implantation occurs too low in the uterus, near or covering the cervical opening, while placenta PAS disorders result from abnormally deep trophoblast, where the placenta penetrates beyond the endometrium into the myometrium (accreta), reaches the serosa (increta), or invades adjacent organs (percreta). These conditions are potentially life-threatening, as the abnormal implantation patterns disrupt the delicate balance between maternal and fetal tissues, preventing the normal physiological separation of the placenta after delivery and are often associated with massive obstetric hemorrhage which may require urgent hysterectomy [2].

However, the exact pathogenesis of PAS remains unclear. The prevailing theory suggests a multifactorial origin involving anatomical, molecular, and physiological factors that, in combination, lead to defective placental anchoring, abnormally placenta adherence, and/or impaired vascular remodeling [3, 4]. These implantation abnormalities may associate with pre-existing uterine damage or disrupted interactions between trophoblasts and uterine wall [5]. Despite identifying biomarkers related to PAS in genetic expression pathways for apoptosis, cell proliferation, invasion, and inflammation [6–9], the precise molecular mechanisms remain unclear, which hinders effective prediction and prevention of PAS.

The combined use of metabolomics and transcriptomics provides a more comprehensive understanding of pathophysiology than either method alone [10]. While each approach provides unique insights into biological systems, integrating information from metabolic activity and gene expression will improve our understanding of disease etiology and help uncover the complex mechanisms driving disease development [11, 12]. This method of data integration has been successfully applied in previous studies of obstetrics complications, including preeclampsia and fetal growth restriction, identifying several critical molecular mechanisms [13, 14].

However, research specifically integrating metabolomics and transcriptomics in PAS remains limited. Previous omics-based studies have primarily focused on transcriptome and proteome profiling analyses. For example, a transcriptome and proteome study based on five placenta accreta (PA) cases and controls, identified decreased methyl-CpG-binding domain protein 2, podocin, and apolipoprotein D decreased in PA group-that was decreased in PA tissues, which were associated with increased migration ability of trophoblasts [15]. More recently, a spatial proteomics and transcriptomics demonstrated that deep myometrial invasion in placenta increta regions is characterized by activation of activator protein-1(AP-1) and Nuclear Factor κB (NF-κB) signally, extracellular matrix remodeling, and an immunosuppressive microenvironment [16]. Nevertheless, integrated analyses combining comprehensive metabolomic and transcriptomic profiling to elucidate metabolic reprogramming in PAS are still lacking, highlighting a significantly gap in understanding the metabolic mechanisms underlying abnormal trophoblast adherence.

The aim of the present study is to profile in greater detail the metabolites and gene-expression changes that occur in placenta of pregnant women with PAS, hence providing mechanisms driving abnormal placental development in PAS and the potential biomarkers for it.

## Materials and methods

### Sample collection

Placental tissues were obtained immediately following cesarean delivery at Qilu Hospital, Shandong University (Jinan, China). Control placental tissues were obtained from women with uncomplicated pregnancies and were matched to PAS cases based on gestational age at delivery.

This study concluded two cohorts, a discovery cohort for multi-omics analyses and a validation cohort. Due to the strict tissue quality requirements for metabolomics and transcriptomics analyses as well as the clustering of PAS cases during the collection period, 19 PAS and 10 control samples were ultimately included in the discovery cohort for integrated multi-omics analysis. To validate key pathway-related findings identified in the discovery cohort, an expanded cohort was subsequently analyzed, including 22 PAS and 50 control samples.

PAS was diagnosed based on FIGO 2019 criteria for PAS and prenatally using an established ultrasound-based scoring system (a score ≤ 5 predicted only accreta type, a score 6–10 (≥ 6 and < 10) predicted placental increta, a score ≥ 10 suggesting placenta precreta) [17], and confirmed intraoperatively by at least two senior obstetricians with extensive PAS operation experience. The ultrasound images of several typical cases of placental implantation were shown in Fig. S1. The available intraoperative images of PAS before placental separation were shown in Fig. S2. Histopathological examination was available for all placental specimens. Cases with local resection of the implanted lesion provided histological evidence of abnormal villous implantation (Fig. S3). To minimize misclassification between PAS and severe uterine dehiscence, all cases were strictly re-reviewed using combined imaging and surgical criteria, and non-adherent dehiscence cases were excluded. Placental tissue samples were obtained intraoperatively immediately after delivery from the most representative lesion areas identified by ultrasound and surgical inspection [18].

Tissue samples containing villous and extravillous trophoblasts, the fibrinoid layer, and basal plate were collected from the implantation site, rinsed in saline, snap-frozen in liquid nitrogen, and stored at − 80℃. Exclusion criteria included multiple pregnancy, fetal anomalies, infections, autoimmune diseases, and pregnancy complications such as hypertensive disorders or gestational diabetes. Placenta previa was not used as a matching criterion, as the primary objective was to compare placental tissues with abnormally adherent to those from uncomplicated pregnancies. Written informed consent was obtained from all participants. Ethics approval was granted by the Qilu Hospital Ethics Committee (KYLL-2024-032-1).

### Untargeted metabolomics profiling

Approximately 100 mg of placental tissue was extracted in 80% methanol, vortexed, incubated on ice, centrifuged, and the supernatant subjected to UHPLC-MS/MS (Ultra-High-Performance Liquid Chromatography–Tandem Mass Spectrometry, Vanquish system coupled with Q Exactive HF, Thermo Fisher). Data acquisition was performed in both ionization modes.

### Data processing and metabolite identification

Raw UHPLC–MS/MS data files were processed using Compound Discoverer 3.3 (CD3.3, Thermo Fisher Scientific) for peak alignment, peak detection, and metabolite quantification. The main parameters were set as follows: mass tolerance of 5 ppm, signal intensity tolerance of 30%, and a predefined minimum intensity threshold. Peak areas were corrected based on the first quality control sample (QC1) to minimize analytical variability. Background ions were removed using blank samples.

Peak intensities were normalized to total spectral intensity. For samples with non-normally distributed data, relative peak areas were further calculated using the following normalization formula:

$$\frac{\text{sample raw quantification value}}{\text{total metabolite intensity of the sample / total metabolite intensity of QC1}}$$

To ensure analytical reproducibility, metabolites with a coefficient of variation (CV) greater than 30% in QC samples were excluded from subsequent analysis.

Molecular formulas were predicted based on adduct ions, molecular ion peaks, and MS/MS fragment ions. Metabolite identification was achieved by matching accurate mass measurements and fragmentation spectra against the mzCloud (https://www.mzcloud.org/), mzVault, and MassList databases, generating qualitative and relative quantitative results.

### Data analysis

Metabolites were further annotated using the KEGG (Kyoto Encyclopedia of Genes and Genomes, https://www.genome.jp/kegg/pathway.html), HMDB (https://hmdb.ca/metabolites), and LIPID MAPS databases (http://www.lipidmaps.org/) for functional interpretation.

Principal component analysis (PCA) was performed to evaluate overall metabolic variation and sample distribution among groups. QC samples were included in the PCA to assess analytical stability.

Univariate statistical analysis was conducted using Student’s t-test to evaluate differences between groups. To control for multiple comparisons, P-values were adjusted using the Benjamini–Hochberg false discovery rate (FDR) method. Metabolites with adjusted *p* < 0.05 and fold change (FC) ≥ 2 or ≤ 0.5 were considered significantly differential metabolites.

Volcano plots were generated in R based on log2-transformed fold changes and -log10-transformed adjusted *p* -values. For hierarchical clustering analysis, intensity data of differential metabolites were standardized using Z-score normalization and visualized as heatmaps.

Pearson correlation analysis was performed to assess associations among differential metabolites, and statistical significance was determined accordingly. Correlation matrices were visualized using R-based graphical tools.

Pathway enrichment analysis was conducted based on the KEGG database. Enrichment significance was determined by comparing the proportion of differential metabolites assigned to a given pathway with the proportion of all identified metabolites mapped to that pathway. Pathways with adjusted *p* < 0.05 were considered significantly enriched. All statistical analyses were performed using R version 3.4.3 in a Linux environment (CentOS release 6.6).

### Transcriptomic profiling

Total RNA from PAS and control placentas was extracted and quality-checked (NanoDrop, Bioanalyzer). RNA-seq libraries were prepared using Illumina protocols and sequenced (150 bp paired-end). Reads were aligned to the human genome (Hierarchical Indexing for Spliced Alignment of Transcripts 2, HISAT2), gene counts obtained (FeatureCounts) and normalized as FPKM (Fragments Per Kilobase of transcript per Million mapped reads). DEGs (Differentially Expressed Genes) were identified using DESeq2 and edgeR (adjusted *p* < 0.05), with GO and KEGG enrichment performed using ClusterProfiler and GSEA (Gene Set Enrichment Analysis).

### RT-qPCR validation

Expression of key genes in the phosphatidylcholine pathway was validated by RT-qPCR (SparkJade, China). cDNA was synthesized and amplified using SYBR Green chemistry. GAPDH was used as the internal control. Relative expression was calculated using the 2^–ΔΔCt^ method. The sequences of primers used are listed in Supplementary Table S1.

### ELISA

Lecithin levels in placental tissue were measured using a human Lecithin ELISA kit (Shanghai Kaibo Biotechnology Co., Ltd.). Briefly, 100 mg of tissue was homogenized in 1 mL PBS (pH 7.4) and centrifuged at 3000 rpm for 20 min. Standards (0–80 µmol/L) and samples were added to a 96-well plate, followed by 100 µL of enzyme conjugate per well, except for blank wells. After sealing, the plate was incubated at 37 °C for 60 min. Wells were washed five times with washing buffer, then 50 µL each of Chromogen A and B was added and incubated in the dark at 37 °C for 15 min. The reaction was stopped with 50 µL stop solution, and absorbance was read at 450 nm.

### Statistical analysis

Continuous data were expressed as mean ± SD or median (IQR), and compared using Welch’s t-test or Mann–Whitney U test, as appropriate. Categorical variables were analyzed using chi-square or Fisher’s exact test. *P* < 0.05 was considered statistically significant. Receiver operating characteristic (ROC) curve analysis was performed to assess the diagnostic performance of PCYT1B, Lecithin, and their combination for PAS diagnosis. The area under the curve (AUC), sensitivity, specificity, and Youden index were calculated to compare discriminative abilities. The DeLong test was used to compare AUCs. Analyses were performed using SPSS v27.0 (IBM Corp., Armonk, NY, USA).

## Results

### Descriptive characteristics and infant outcomes

PAS cases and control cases were included for metabolomics and transcriptomics analyses in the study. The maternal demographic characteristics and infant outcomes of the two groups are presented in Table 1. There were no significant differences in maternal age, BMI (Body Mass Index), and gestational age at delivery, between the PAS and control groups. Importantly, neonatal birth weight also comparable between groups, suggesting that placentas affected by PAS may have minimal impact on fetal growth. The PAS group had significantly higher gravidity (*p* = 0.014), increased incidence of blood loss ≥ 1000 mL (*p* = 0.012) and a markedly higher rate of previous cesarean sections compared to controls (*p* < 0.0001). Implantation scores were available only for the PAS group, with 52.63% of patients scoring from 6 to 10 and 42.11% scoring above 10.

**Table 1:** Comparison of clinical characteristics between PAS and control groups (CON)

Variable	PAS Group (*n* = 19)	Control Group (*n* = 10)	*p* -value
Maternal Age (y)			0.454
	34.16 ± 1.12	32.70 ± 1.58	
BMI(kg/m^2^)			0.272
	28.21 ± 0.72	26.75 ± 1.18	
Gravidity *n* (%)			0.014*
1–2	3 (15.79%)	6 (60%)	
≥ 3	16 (84.21%)	4 (40%)	
History of intrauterine surgery^△^ ,*n *(%)			0.009*
	19 (100%)	4 (40%)	
Placenta previa in current pregnancy, *n* (%)			< 0.001*
	19 (100%)	2 (20%)	
Implantation Score^#^ *n* (%)			—
≤ 5 points	1 (5.26%)	—	
6–10 points	10 (52.63%)	—	
≥ 10 points	8 (42.11%)	—	
Gestational Age at delivery(weeks)			0.190
	36.21 ± 0.36	37.45 ± 0.82	
Neonatal Birth Weight(g)			0.951
	2651.58 ± 100.22	2640.50 ± 157.05	
Intraoperative Blood Loss^△△^ (ml)			0.012*
≤ 1000	10 (52.63%)	10 (100%)	
> 1000	9 (47.37%)	0 (0%)	

*BMI* body mass index calculated as weight in kilograms divided by height in metres squared.*Bold number: significant difference. ^#^ Implantation score calculated using an ultrasonic scoring system which is widely used in China [17]. ^△^ Intrauterine surgery includes C-section, dilation and curettage, myomectomy, hysteroscopic procedures, etc. ^△^^△^ including the volume of autologous blood transfusion

### Non-targeted metabolomics analysis of metabolites alteration in placental tissues from patients with PAS

**Fig. 1:**
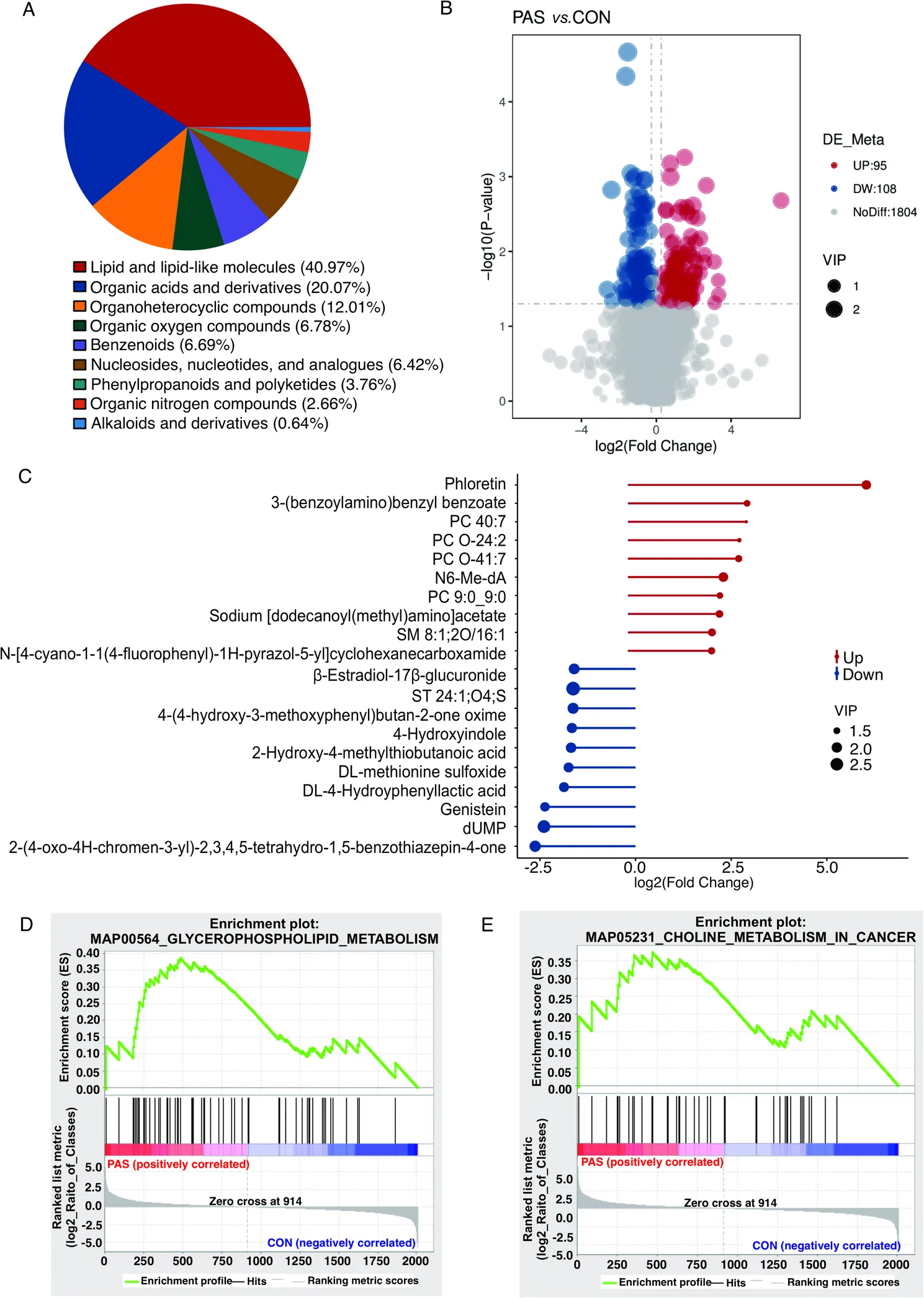
Non-targeted metabolomics analysis of metabolites alteration in placental tissues from patients with PAS comparing with control groups (CON). **A** Classification of detected metabolites in placenta samples, showing the relative proportion of major metabolic categories. **B** Volcano plots displaying 203 differentially expressed metabolites (DEMs) between PAS and control groups. The x-axis represents the fold change of metabolites between different groups (log2 (Fold Change)), while the y-axis indicates the significance level (-log10(*P*-value)). Significantly upregulated, downregulated (FC > 2 or FC ≤ 0.5, and *p*-value < 0.05) and non-significantly changed metabolites are colored in red, blue and grey, respectively. The size of the dots corresponds to the VIP (Variable Importance in Projection) values. **C** Stick plot ranking the top ten upregulated and top ten downregulated metabolites ranked by log2 fold change. Dots are colored in red and blue, represent upregulation and downregulation, respectively. The length of the bars represents the magnitude of log2 (fold change), while the size of dots represents the VIP value. **D** Gene set enrichment analysis (GSEA) enrichment plot for glycerophospholipid metabolism (KEGG pathway MAP00564). **E** GSEA enrichment plot for choline metabolism in cancer (KEGG pathway MAP05231)

Untargeted metabolomics analysis of placental tissues from PAS and control pregnancies identified 203 differential metabolites (95 upregulated, 108 downregulated), with lipids and lipid-like molecules comprising the largest category (40.97%), followed by organic acid and derivatives (20.07%) (Fig. 1A-B). Notably, several phosphatidylcholine (PC) species (PC 40:7, PC O-24:2, PC O-41:7 and PC 9:0_9:0) were significantly elevated in PAS (Fig. 1C), implicating dysregulated glycerophospholipid metabolism. Notably, glycerophospholipid metabolism (KEGG ID: MAP00564) and choline metabolism in cancer (KEGG ID: MAP05231) was enriched in PAS (Fig. 1D and E). The detailed results of GSEA enriched terms for these two metabolic pathways were provided in Table S2. To explore the overall variation in metabolic profiles between PAS and control samples, principal component analysis (PCA) was also performed to visualize overall metabolic differences between groups. The first three principal components (PC1: 18.8%, PC2: 12.2%, PC3: 8.8%) together explained 39.8% of the total variance. The score plot showed a partial separation trend between groups, indicating underlying metabolic distinctions. (Fig. S4).

### Transcriptome analysis reveals differential gene expression and disease associations in PAS

**Fig. 2:**
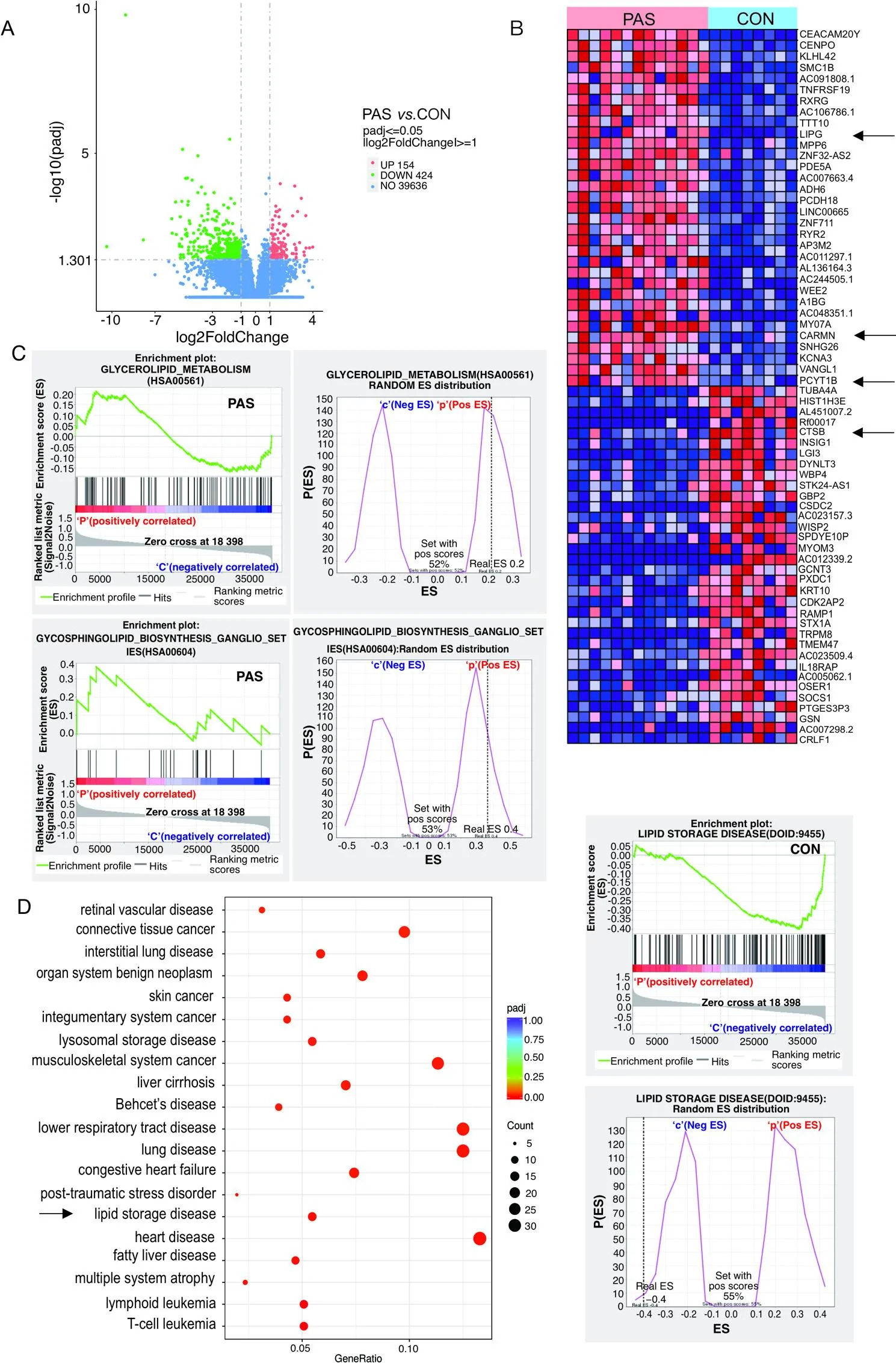
Differential gene expression and disease enrichment analysis. **A** Volcano plot displaying differentially expressed genes (DEGs) between PAS and control groups. The x-axis represents log2 (Fold Change), while the y-axis represents -log10 (adjusted *p*-value). Genes significantly upregulated (log2 Fold Change ≥ 1, adjusted *p*-value (padj) ≤ 0.05) are marked in red, while significantly downregulated genes are in green. Non-significant genes are shown in blue. **B** Heatmap of the top differentially expressed genes, with PAS and control groups labeled at the top. Red represents high expression, while blue indicates low expression. Selected DEGs with significant changes are marked with arrows. **C** Gene Set Enrichment Analysis (GSEA) plots for PAS and CON groups after KEGG analysis. The top left and bottom left panels show enrichment plots for “GLYCEROLIPID_METABOLISM (hsa00561)” and “GLYCOSPHINGOLIPID_BIOSYNTHESIS_GANGLIO_SERIES (hsa00604)” in PAS. The top right and bottom right panels show the distribution of random enrichment scores (ES) for the respective pathways. The enrichment score profile, hit positions, and ranking metric scores are shown. **D** Disease ontology (DO) enrichment analysis of disease terms associated with DEGs. The y-axis lists enriched diseases, and the x-axis represents GeneRatio (the proportion of DEGs involved in each disease). The size of the dots corresponds to the number of DEGs, and color represents the adjusted *p*-value (padj), with red indicating higher significance. “Lipid storage disease” is marked with an arrow for emphasis. GESA plots for lipid storage disease in CON groups was showed on the right

Transcriptomic analysis identified 578 differentially expressed genes (154 upregulated, 424 downregulated), including upregulation of lipase G (LIPG, *p* = 0.001) and phosphate cytidylytransferase 1B (PCYT1B, *p* = 0.016), which are involved in lipid metabolism (Fig. 2A-B). Gene Set Enrichment GSEA showed enrichment of glycerolipid metabolism and glycosphingolipid biosynthesis pathways in PAS (Fig. 2C). Disease ontology analysis linked DEGs to lipid storage diseases which showed negative enrichment GSEA in controls placenta further supporting metabolic dysregulation (Fig. 2D). 

### Integrated multi-omics analysis reveals enhanced phosphatidylcholine biosynthesis in PAS placenta

**Fig. 3:**
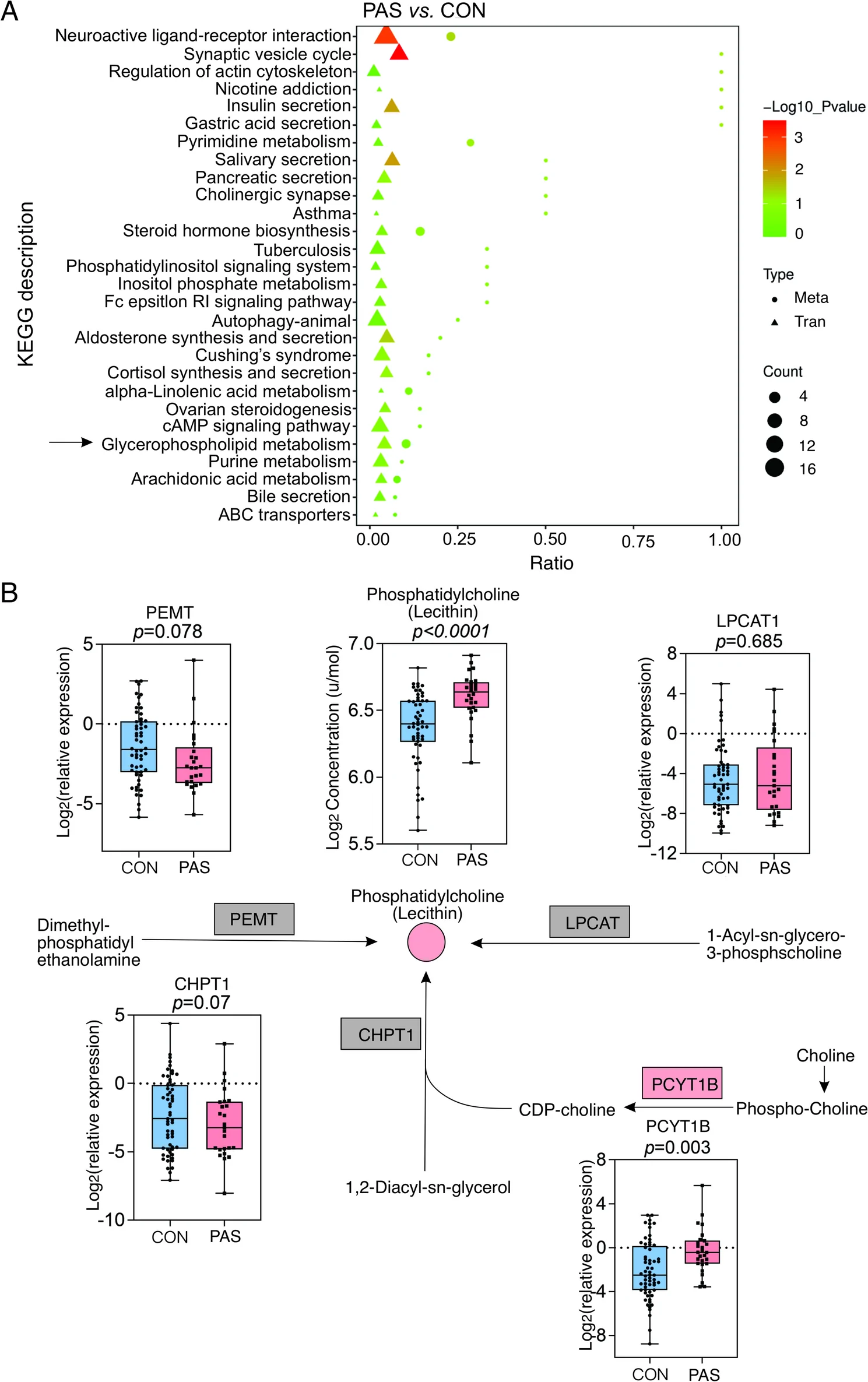
Enhanced phosphatidylcholine biosynthesis in PAS placenta by muti-omics analysis. **A** KEGG pathway enrichment analysis based on integrated metabolomic and transcriptomic data (PAS vs. CON). The x-axis represents the ratio of differential metabolites or genes enriched in a given pathway to the total number of metabolites or genes annotated in that pathway (Ratio). Dot size indicates the number of genes/metabolites involved; color indicates *p*-value (− log10-transformed). Triangles represent transcriptomic data; circles represent metabolomic data. **B** Schematic representation of the phosphatidylcholine biosynthesis pathway, with gene expression changes (Log2 relative expression) for PEMT, LPCAT1, CHPT1, PCYT1B and phosphatidylcholine (lecithin, Log2 concentration) shown in PAS (*n* = 22, red box) and CON (*n* = 50, blue box). Illustration shows CDP-choline pathways that achieved statistically significantly increased (red) and non-significantly changed (grey). The box plots are visualized as median and 25^th^ and 75^th^ percentiles, with whiskers indicating maximal and minimal values. PEMT: phosphatidylethanolamine N-methyltransferase; LPCAT1: Lysophosphatidylcholine Acyltransferase 1. CHPT1: Choline Phosphotransferase 1; PCYT1B: choline-phosphate cytidylyltransfease1B

Integrated metabolomic-transcriptomic analysis highlighted glycerophospholipid metabolism as a key dysregulated pathway (Fig. 3A). To validate these findings, an expanded cohort was analyzed. In this validation cohort, PCYT1B was significantly upregulated (*p* = 0.003), while CHPT1(Choline Phosphotransferase 1), LPCAT1 (Lysophosphatidylcholine Acyltransferase 1), and PEMT(Phosphatidylethanolamine N-Methyltransferase) showed no significant differences. Additionally, total phosphatidylcholine (Lecithin) levels were markedly elevated in PAS placentas (*p* < 0.0001), suggested increase in the CDP-choline pathway activity (Fig. 3B). Baseline characteristics between groups were comparable (Table S3), except for a little higher maternal age (*p* = 0.031) and increased incidence of blood loss ≥ 1000 mL in PAS cases. When PAS patients were stratified into three subgroups (score ≤ 5, score 6–10, and score ≥ 10), lecithin expression was significantly elevated in the score 6–10 and ≥ 10 groups compared with the control group. For PCYT1B, only the ≥ 10 group showed significantly higher expression compared with controls. CHPT1 exhibited differences between the ≤ 5 group and both the control and score 6–10 groups, although interpretation is limited given the small sample size (*n* = 3) in the ≤ 5 group. PMET and LPCAT1 did not show significant differences across subgroups (Fig. S5). To integrate these findings and illustrate the potential mechanistic implications, we constructed a schematic model summarizing the altered PC metabolism in PAS (Fig. S6). In this proposed model, increased PC levels may reflect enhanced membrane biogenesis, which could be associated with abnormal placental adherence and placental attachment.

### ROC curve analysis of Lecithin, PCYT1B, and their combination for PAS diagnosis

**Fig. 4:**
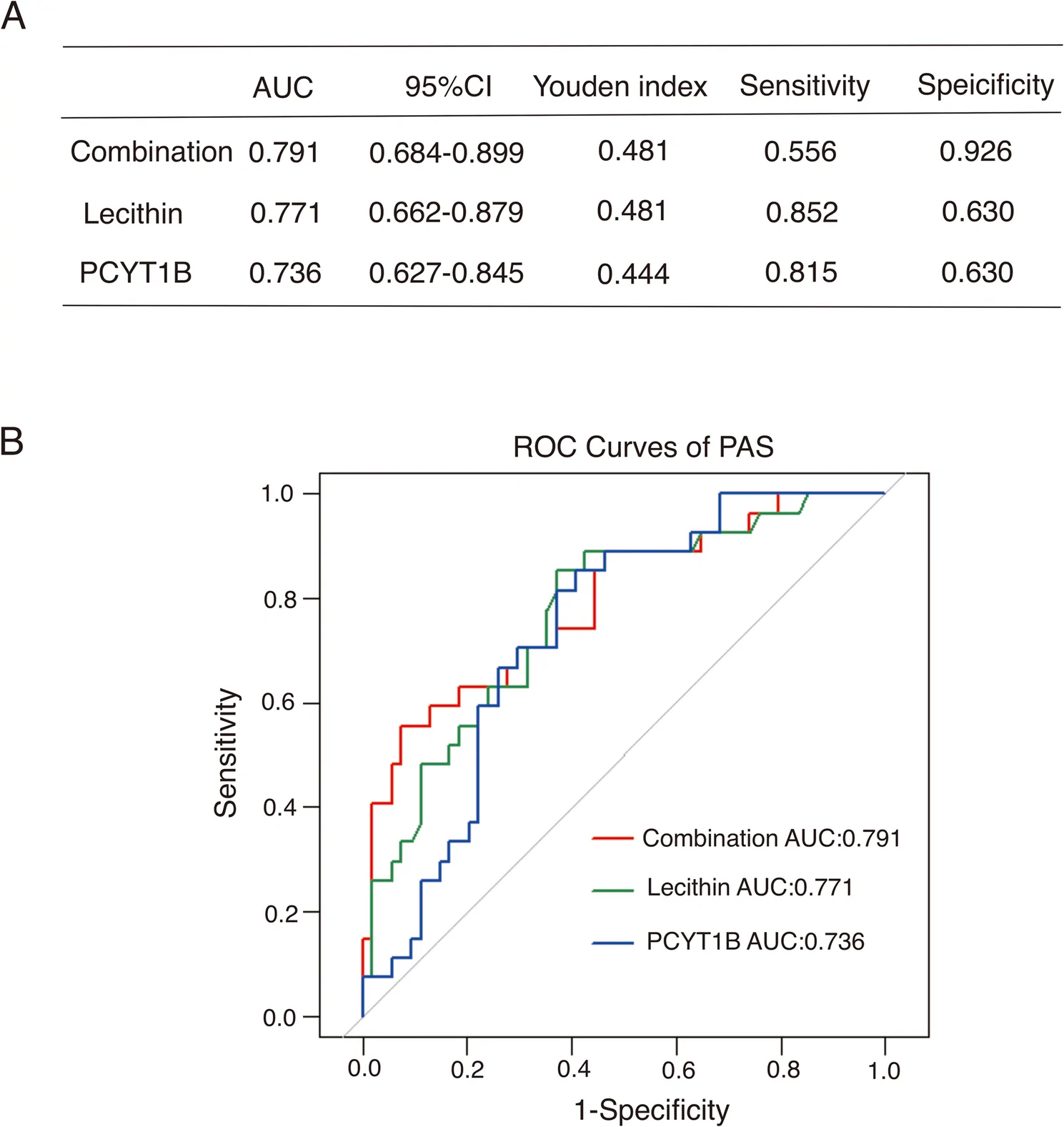
Diagnostic performance of Lecithin, PCYT1B, and their combination for predicting PAS. **A** Sensitivity, specificity, and Youden index for each marker and the combination. **B** ROC curves showing discriminative ability. AUC values are shown on the curves

ROC curve analysis was conducted to evaluate the diagnostic performance of PCYT1B, Lecithin, and their combination in diagnose PAS (Fig. 4). Lecithin alone showed higher sensitivity (85.2%) but lower specificity (63.0%), yielding the Youden index of 0.481, whereas PCYT1B alone demonstrated intermediate performance with a Youden index of 0.444 (sensitivity 81.5%, specificity 63.0%).The combination of PCYT1B and Lecithin achieved the best discriminative ability with an AUC of 0.791 (95% CI: 0.684–0.899) and the Youden index of 0.481 (lower sensitivity 55.6%, higher specificity 92.6%).

## Discussion

In this integrated metabolomic and transcriptomic analysis, we provided evidence which may be a potential significant reprogramming of lipid metabolism, particularly in phosphatidylcholine (PC) biosynthesis. These findings expand current understanding of PAS, as previous studies have largely focused on histopathological, immunological, or angiogenic aspects [5, 19, 20].

Specifically, non-targeted metabolomics data identified substantial accumulation of PC species, including PC 40:7, PC O-24:2, and PC O-41:7, in PAS tissues. Although pathway enrichment analysis also indicated alterations in purine, tryptophan, and phenylalanine metabolism; however, these pathways were not the focus in this study. Transcriptomic profiling showed upregulation of PCYT1B, a rate-limiting enzyme in the CDP-choline pathway, which may contribute to PC accumulation in PAS placenta. These findings were further validated in an expanded cohort, supporting the robustness of the observed metabolic signature.

PC enrichment in PAS placentas may be associated with a metabolic adaptation to support excessive trophoblast proliferation, as well as a potential modulation of the inflammatory microenvironment. As the most abundant glycerophospholipid in eukaryotic membranes, PC plays a crucial role in placental development and function [21, 22]. Adequate PC supports placental terminal villi formation in response to maternal perfusion, and help modulate angiogenesis, inflammatory responses and prevent apoptosis in trophoblast [23–25]. Enhanced PC biosynthesis in PAS resembles metabolic patterns in malignancies, where upregulated PC sustains aggressive cellular behavior [26]. Additionally, PCs serve as precursors for lipid mediators such as lysophosphatidylcholine (LPC) and diacylglycerol (DAG), which are known to influence angiogenesis, immune responses, and cell motility [27, 28].

We further evaluated key enzymes involved in the three principal pathways of PC biosynthesis: PCYT1B (the Kennedy pathway), PEMT (the phosphatidylethanolamine PE methylation pathway) and LPCAT (the Lands’ cycle). Notably, only PCYT1B was significantly upregulated in PAS placentas, while PEMT and LPCAT showed no significant changes. We propose a potential mechanistic association between PCYT1B upregulation and PC metabolism in PAS placentas. Upregulated PCY1TB may enhance *de novo* PC synthesis, facilitating membrane biogenesis and remodeling, which is essential for rapidly proliferating and invasive trophoblast [29–31]. In contrast, the PE methylation pathway predominantly functions in the liver to maintain lipid homeostasis, and the Lands’ cycle mediated by LPCAT regulates membrane remodeling, lipid signaling, and stress responses [32, 33]. The selective upregulation of PCYT1B may indicate targeted enhancement of membrane biogenesis in trophoblast of PAS, providing a possible explanation for aberrant placental implantation.

Targeting PC pathway may represent novel intervention strategies. However, based on the current associative data, these alterations are more likely to reflect downstream adaptation rather than direct causative factors. Maternal PC or choline levels could influence placental development, warranting further investigation. Stratified analysis showed that both Lecithin and PCYT1B were elevated in PAS patients with higher ultrasound scores, suggesting a potential association between Kennedy pathway activation and disease severity.

Currently, ultrasound and MRI are widely used for PAS diagnosis. Although standardized imaging criteria have improved diagnosis accuracy, interpretation remains partially subjective and operator dependent. In contrast, metabolite-based biomarkers and the related pathways provide objective and quantifiable measurements. Lecithin alone showed an AUC of 0.771, and combined with PCYT1B achieved an AUC of 0.791, with high specificity (92.6%) but moderate sensitivity (55.6%). This indicates that the model may reliably identify non-PAS cases and could serve as complementary biomarkers to improve risk stratification. Future studies are warranted to evaluate the sensitivity and specificity of these biomarkers in plasma samples and to compare their performance directly with ultrasound and MRI in early detection of PAS.

Our study has limitations. First, patient heterogeneity remains a concern. Although most PAS cases had severe forms (placenta increta and precreta), PAS samples are more widely dispersed in PCA score plot, which potentially introducing analytical bias. Second, histologic confirmation of PAS was not fully available, as all patients undergoing conservative surgery. Third, no functional assays were performed; our conclusion are primarily based on correlative evidence derived from transcriptomic and metabolomic profiling. Finally, the relatively small size for each PAS subtype may compromise statistical power for subtype-specific comparisons. Therefore, the subtype-related findings in this study should be interpreted with caution. Future studies will aim to increase the sample size, achieve a more balanced distribution across PAS subtypes, incorporate histologic confirmation when feasible to strengthen diagnostic accuracy, and experimentally validate the role of CDP-choline pathway in PAS pathogenesis.

In summary, our multi-omics study suggests an association between phosphatidylcholine metabolism alterations and in PAS pathogenesis, improving current understanding of the molecular mechanisms underlying PAS. These findings may help fill the existing knowledge gaps in PAS research and generate hypotheses for subsequent diagnostic and therapeutic strategies. 

## Supplementary Information

Supplementary Material 1: Fig S1. Ultrasound images of several placental implantation cases (score ≥6). The images show typical features of abnormal placental attachment, including loss of the clear zone, presence of placental lacunae, myometrial thinning, and disruption of the uterine-bladder interface. Fig S2. Representative intraoperative images of PAS. Images were obtained before placental separation. Due to intraoperative bleeding and limited surgical exposure, macroscopic features may not fully demonstrate the depth of placental invasion. Nevertheless, these images illustrate macroscopic features suggestive of PAS. The diagnosis of PAS was established based on comprehensive prenatal ultrasound findings and intraoperative confirmation according to established clinical criteria, rather than solely on photographic appearance. Fig S3. Histopathological images of PAS. (A) Early evidence of accreta. Arrows point to intermediate trophoblastic cells (B) Placenta accreta. Arrows point to the villi. Fig S4. Principal component analysis (PCA) score plot of PAS and control groups. Each dot represents one sample, with PAS cases shown in red and controls in blue. The x- and y-axes represent the first (PC1, 18.18%) and second (PC2, 12.22%) principal components, respectively. Ellipses indicate the 95% confidence regions for each group. Fig S5. Expression levels of key metabolites and genes involved in the CDP–choline pathway across control and PAS subgroups stratified by ultrasound scores. Box plots show log2(relative expression) of Lecithin, PCYT1B, CHPT1, PMET, and LPCAT1 among controls (CON), PAS patients with ultrasound score ≤5, score 6-10, and score ≥10. Statistical comparisons were performed using one-way ANOVA with post hoc multiple testing. *p < 0.05, ****p < 0.0001. Fig S6. A schematic model summarizing the altered PC metabolism in PAS. Table S1. The sequences of primers used in this manuscript. Table S2. Detailed results of GSEA enriched terms for glycerophospholipid metabolism (KEGG ID: MAP00564) and choline metabolism in cancer (KEGG ID:MAP05231). Table S3. Comparison of Clinical Characteristics Between PAS and Control Groups used in qPCR.

## Data Availability

The datasets generated and analyzed during the current study are available from the corresponding author on reasonable request.
