# Supplementary material for: Integrated metabolomic and transcriptomic analysis identifies enhanced phosphatidylcholine metabolism in placenta accreta spectrum
Source: BMC Pregnancy Childbirth. 2026 Jun 23;26:1154. doi: 10.1186/s12884-026-09552-w (PMC13660257; doi:10.1186/s12884-026-09552-w)
Supplement: Supplementary file 1 [file 12884_2026_9552_MOESM1_ESM.zip › Revise 2Legends for supplemetary table and figure.docx]

**Table S1. The sequences of primers used in this manuscript.**

**Table S2. Detailed results of GSEA enriched terms for glycerophospholipid metabolism (KEGG ID: MAP00564) and choline metabolism in cancer (KEGG ID:MAP05231).**

ID: metabolite identifier; Name: metabolite name; RANK IN LIST: position of the gene/metabolite in the ranked list; RANK METRIC SCORE: score assigned by GSEA to rank features; RUNNING ES: running enrichment score across the ranked list; CORE ENRICHMENT: indicates whether the feature is part of the leading-edge subset contributing most to the enrichment signal.

**Table S3. Comparison of Clinical Characteristics Between PAS and Control Groups used in qPCR.**

BMI = body mass index calculated as weight in kilograms divided by height in metres squared. *Bold number: significant difference. ^#^ Implantation score calculated using an ultrasonic scoring system which is widely used in China, that is, a cutoff score of ≥5 was utilized to predict adhesion type and severe forms of placenta accreta spectrum (including placenta increta and percreta). When the score reached ≥10, the probability of placenta percreta was substantially elevated (19).

**Figure S1. Ultrasound images of several placental implantation cases (score ≥6).**
The images show typical features of abnormal placental attachment, including loss of the clear zone, presence of placental lacunae, myometrial thinning, and disruption of the uterine-bladder interface.

**Figure S2. Representative intraoperative images of PAS.**

Images were obtained before placental separation. Due to intraoperative bleeding and limited surgical exposure, macroscopic features may not fully demonstrate the depth of placental invasion. Nevertheless, these images illustrate macroscopic features suggestive of PAS. The diagnosis of PAS was established based on comprehensive prenatal ultrasound findings and intraoperative confirmation according to established clinical criteria, rather than solely on photographic appearance.

**Figure S3. Histopathological images of PAS.**

(A) Early evidence of accreta. Arrows point to intermediate trophoblastic cells (B) Placenta accreta. Arrows point to the villi .

**Figure S4.Principal component analysis (PCA) score plot of PAS and control groups.**
Each dot represents one sample, with PAS cases shown in red and controls in blue. The x- and y-axes represent the first (PC1, 18.18%) and second (PC2, 12.22%) principal components, respectively. Ellipses indicate the 95% confidence regions for each group.

**Figure S5. Expression levels of key metabolites and genes involved in the CDP–choline pathway across control and PAS subgroups stratified by ultrasound scores.**
Box plots show log2(relative expression) of Lecithin, PCYT1B, CHPT1, PMET, and LPCAT1 among controls (CON), PAS patients with ultrasound score ≤5, score 6-10, and score ≥10. Statistical comparisons were performed using one-way ANOVA with post hoc multiple testing. **p* < 0.05, *****p* < 0.0001.

**Figure S6. A schematic model summarizing the altered PC metabolism in PAS**
